# Ethnobotany, Phytochemistry, and Biological Activities of the Genus Cordyline

**DOI:** 10.3390/biom13121783

**Published:** 2023-12-12

**Authors:** Romuald Tematio Fouedjou, Bienvenu Tsakem, Xavier Siwe-Noundou, Hervet P. Dongmo Fogang, Aphalaine Tiombou Donkia, Beaudelaire Kemvoufo Ponou, Madan Poka, Patrick H. Demana, Rémy B. Teponno, Léon Azefack Tapondjou

**Affiliations:** 1 Research Unit of Environmental and Applied Chemistry, Department of Chemistry, Faculty of Science, University of Dschang, Dschang P.O. Box 67, Cameroon; tematioromy@yahoo.fr (R.T.F.); tsakemb@yahoo.fr (B.T.); atiombou@yahoo.com (A.T.D.); beaudelaireponou@yahoo.fr (B.K.P.); tapondjou2001@yahoo.fr (L.A.T.); 2 Department of Pharmaceutical Sciences, School of Pharmacy, Sefako Makgatho Health Sciences University, P.O. Box 218, Pretoria 0208, South Africa; madan.poka@smu.ac.za (M.P.); patrick.demana@smu.ac.za (P.H.D.); 3 Department of Physiological Sciences and Biochemistry, Faculty of Medicine and Biomedical, Sciences, University of Garoua, Garoua P.O. Box 317, Cameroon; hervpaulain@yahoo.fr

**Keywords:** *Cordyline*, ethnopharmacology, phytochemistry, biological activities

## Abstract

*Cordyline* species have a long history in traditional medicine as a basis of treatment for various ailments such as a bloody cough, dysentery, and a high fever. There are about 26 accepted species names in this genus distributed worldwide, including *C. fruticosa*, *C. autralis*, *C. stricta*, *C. cannifolia*, and *C. dracaenosides*. This work presents a comprehensive review of the traditional uses of plants of the genus *Cordylie* and their chemical constituents and biological activities. A bibliographic search was conducted to identify available information on ethnobotany, ethnopharmacology, chemical composition, and biological activities. A total of 98 isolated compounds potentially responsible for most of the traditional medicinal applications have been reported from eight species of *Cordyline* and are characterised as flavonoid, spirostane, furostane, and cholestane glycosides. Some of these pure compounds, as well as extracts from some species of *Cordyline*, have exhibited noteworthy anti-oxidant, antiproliferative, antimicrobial, and hypolipidemic activities. Although many of these species have not yet been investigated phytochemically or pharmacologically, they remain a potential source of new bioactive compounds.

## 1. Introduction

Traditional medicine frequently relies on medicinal plants for the treatment of various ailments and diseases. In developing countries, traditional medicine plays a significant role in delivering primary health care. Scientists have relied on the wealth of the floral kingdom for nearly half of all new drug discoveries to date [1,2]. Even ornamental plant species can contain a vast wealth of bioactive compounds valuable to medicine [3]. For example, drugs currently in clinical use for cancer treatment include paclitaxel, derived from *Taxus brevifolia* L., vincristine, derived from *Catharanthus roseus* (L.) G. Don., and epigallocatechin-3-gallate, a phenolic catechin from *Camellia sinensis* [4], all of which are better known as ornamental rather than medicinal plants.

Natural products and their derivatives are a significant source for novel drug development in modern medicine, and many of these derivatives form the active ingredient of drugs approved by the U.S. Food and Drug Administration (FDA) [5]. Dependency on direct medicinal plant derivatives is particularly marked in developing countries, where western medicine is often absent or simply too expensive. Plants of the genus *Cordyline*, well known as ornamental plants, have long been used as a source of traditional medicines and are of great interest in primary health care. As with *Taxus brevifolia*, *Catharanthus roseus,* and *Camelia sinensis*, these plants could be highly significant for drug discovery.

According to the literature, *Cordyline* species are characterised by the presence of flavonoids and saponins, particularly spirostane, furostanes, and cholestane glycosides, with various structural skeletons [6,7,8]. These compounds have been reported to show a wide range of biological activities, including anti-inflammatory, antiproliferative, antimicrobial, cytotoxic, and hypoglycaemic properties [7,9,10,11]. Some activities of these plants have been investigated, prompted by their anecdotal usage in traditional medicine, and various biological activities such as antidiabetic, anti-ulcer, antidiarrheal, wound healing, and anti-inflammatory properties have been reported in several scientific publications [7,12,13,14].

Despite literature surveys dating back to 1984, no reviews have summarised the ethnomedicinal uses, phytochemical constituents, and biological activities of plants of the genus *Cordyline*. Accordingly, this review provides an up-to-date synopsis of all bioactive compounds isolated from different parts of *Cordyline* species, as well as their biological activities and ethnopharmacological uses. This information is intended to summarise the therapeutic potential and to help guide future research. Databases such as Scopus, ScienceDirect, PubMed, and Google Scholar were used to source scientific articles. From ancient times up to 2022, eight species have been studied, and 56 saponins, 20 flavonoids, and 20 sapogenins were isolated and characterised from these plants. We have included in this paper the reported biological activities of some of these isolated compounds, as well as the activities of some extracts.

## 2. Botany and Taxonomy

**Figure 1:**
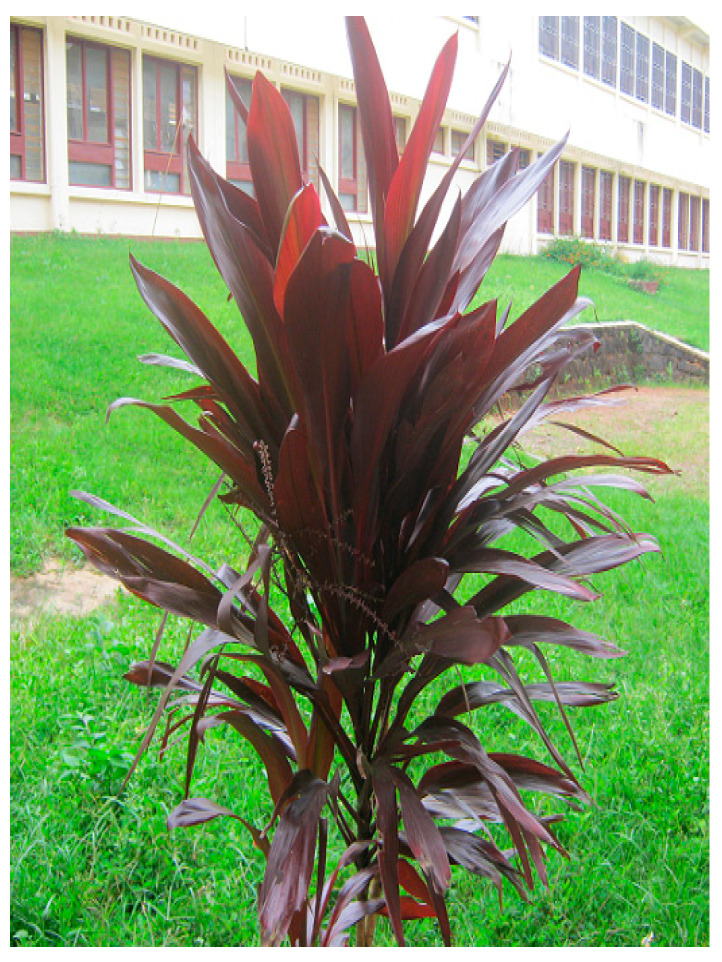
*Cordyline fruticosa*.

The name *Cordyline* is derived from the Greek *kordyle*, which means *club*, which refers to the plants’ thick underground stems or rhizomes. Royen was the first to report the name *Cordyline* in 1740 and to propose a classification, but due to the presence of *Yucca* and *Dracaena* in his classification, Adanson gave the plant a new generic classification in 1763 [15]. The genus *Cordyline,* one of the so-called ‘tree lilies’, was placed in the family Laxmanniaceae according to the Angiosperm Phylogeny Group (APG) in 2003, then in the Asparagaceae family in 2009. Also called Agavaceae, this family contains 128 genera, with more than 3000 species [16]. The genus *Cordyline* contains 26 species alone, of which *C. fruticosa, C. australis, C. stricta*, *C. annifolia*, *C. rubra,* and *C. dracaenoside* appear to have been the most researched. *Cordyline australis*, commonly called the cabbage tree and known as ‘ti kouka’ by the Maori people, is the most widespread of this genus and occurs in open places and at forest margins on the three main islands [17]. *Cordylines* form small trees or shrubs and have leathery leaves, which are often clustered and palm-like in appearance. *Cordyline* species have creamish white roots that often form root suckers starting from their rhizomes [18,19,20]. *Cordyline* belongs to the few genera of monocots in which a cambium is present in the stem. The most well-known ornamental and medicinal plant of this genus is *Cordyline fruticosa* L. (Chev.) (Figure 1), with its beautiful and attractive decorative foliage.

Plants of the genus *Cordyline* generally reach up to 3 m high and are remarkable for their colourful foliage and varied heights, colours, and the shape of their leaves [21,22,23]. Several hybrid combinations of the *Cordyline* species have been recorded. The species is believed to have originated in Southeast Asia and Papua New Guinea. The plants of the genus *Cordyline* are not always easy to distinguish from those of the *Dracaena*. However, the petiole of the former is generally larger than that of the latter (10 to 30 cm versus 1 to 8 cm).

## 3. Traditional Uses of the Genus *Cordyline*

Several plants of the genus *Cordyline*, and particularly their leaves, are used in traditional medicines throughout the tropical and subtropical regions for the treatment of various diseases such as dysentery, skin infection, rheumatism, inflammations, and periodic fever [24,25].

*Cordyline dracaenoides* is used as a traditional medicine in the south of Brazil as an anti-inflammatory preparation for the treatment of rheumatoid and related diseases [26]. *Cordyline stricta* is an evergreen shrub native to Australia, and an extract of its leaves is used as a hemostatic in traditional Chinese medicine [9]. *Cordyline fruticosa*, commonly known as the good luck plant, is grown in tropical and subtropical regions as an ornamental plant. In Polynesia, it is used for both cultural purposes and as a food crop [27]. The plant is widely utilised in both everyday life and as a source of traditional medicine. The leaves are used for costumes, decorations, clothing, sandals, packaging, and cooking. The rhizomes can be baked into a molasses-like food product and eaten [28]. In addition, the plant is used for the treatment of various diseases. The leaves are used to treat sore throat and neck pain [29], as a hemostatic [30,31], and to induce abortion [32], and the roots are used for toothache, laryngitis, and to treat infections of the mammary glands [31,33].

In Sabah, the Kadazan-Dusun people, who are the largest ethnic group in Malaysia, use the roots of *C. terminalis* L. Kunth (also known as *C. fruticosa*) (locally called *Pipisokalaganan*) as part of their local medicine for curing cough, bloody cough, dysentery, high fever, difficulties in urinating, bloody urine, kidney diseases, headaches, inflammation in the digestive tract, scurf, and joint pain. For example, the roots are pounded into a paste and applied to the stomach to relieve stomach pain [34]. In Hawaii, local people use the purple flowers of *C. fruticosa* to treat asthma and growths in the nose [35].

## 4. Other Important Uses of Plants of the Genus *Cordyline*

Ornamental plants are characterized by attractive foliage and/or flowers that can survive and grow indoors and/or outdoors. *Cordyline fruticosa*, with its attractive red decorative foliage, is one of India's most economically important ornamental houseplants [36]. The economic value of ornamental plants of the genus *Cordyline* has risen perceptibly worldwide and is still increasing to meet the steady demand of the floriculture industry. Reports that the roots of *Cordyline australis* may contain more than 60% fructofuranan suggest that the species could become a commercial source of fructose [37]. *Cordyline australis* has been used extensively by the Maori people in Australia, both as a source of fibre and as a food, and there is strong evidence to suggest that the plant was translocated extensively in pre-European times [38].

*Cordyline fruticosa* is often used ornamentally as a low-growing border shrub [39]. *C. fruticosa* is associated with cultural, material, and religious uses among the peoples of Oceania [18,40]. There is some evidence that the early Polynesians used *Cordyline fruticosa* for its importance in costume making, for wrapping food, for religious uses, and as a food source [41]. A hedge of *C. fruticosa* around the house was believed to ward off evil spirits and bring good luck [42]. The fleshy rhizome contains up to 20% sugar, mainly fructose, and is used as a natural sweetener in New Zealand and for the production of an alcoholic beverage in Hawaii [43]. In Hawaii, the fibre from the leaves of the ‘Ti’ plant (a common name for any plant of the genus in Hawaii) was used for making sandals, baskets, bird snares, sieves, and thatch for roofs, rope, and cord. The leaves of the plant (known as ‘rau ti’ in the Cook Islands) or the tender young shoots were eaten raw or roasted in the embers of a fire. Pith (commonly called Ti) was dried in the sun and cooked to make porridge [43]. Roots of Ti were used for making sweet drinks. Sugar was extracted by cooking the roots in an earth oven called an ‘umu-ti’. Large pits were used to steam the roots. Long hedges of *Cordyline fruticosa* are planted by locals to guard the gardens against pigs. The handling of ‘Ti’ was often accompanied by ceremonial protocols [43].

The New Zealand cabbage tree, known as *Cordyline australis*, is internationally rated as one of their most famous indigenous plants. Its popularity is based not only on its characteristic habit, shape, and deliciously fragrant flowers but also on the relatively recent progress of coloured foliage forms [44]. Other species, also known as cabbage trees, include *C. kaspar, C. indivisa* (*Ti toi*), *C. banksii* (*Ti ngahere*), and *C. pumilio* (*Ti rauriki*) [45].

## 5. Chemical Constituents

**Figure 2:**
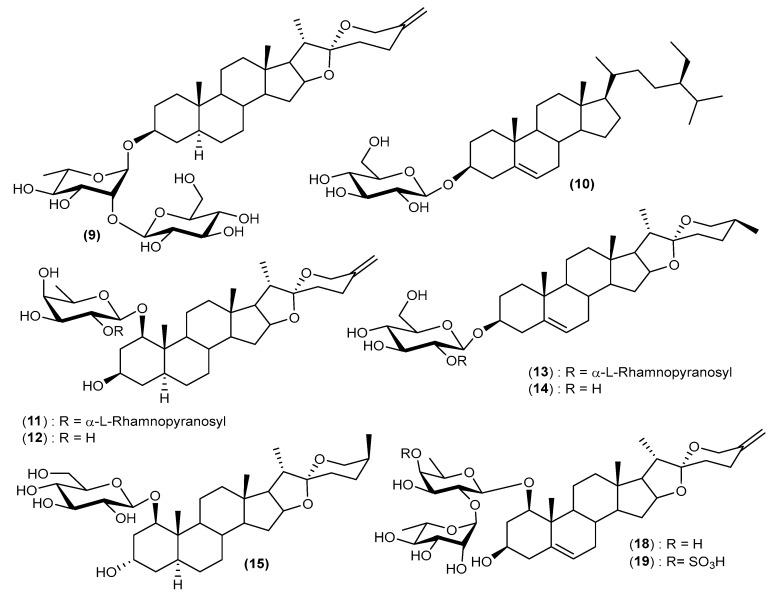

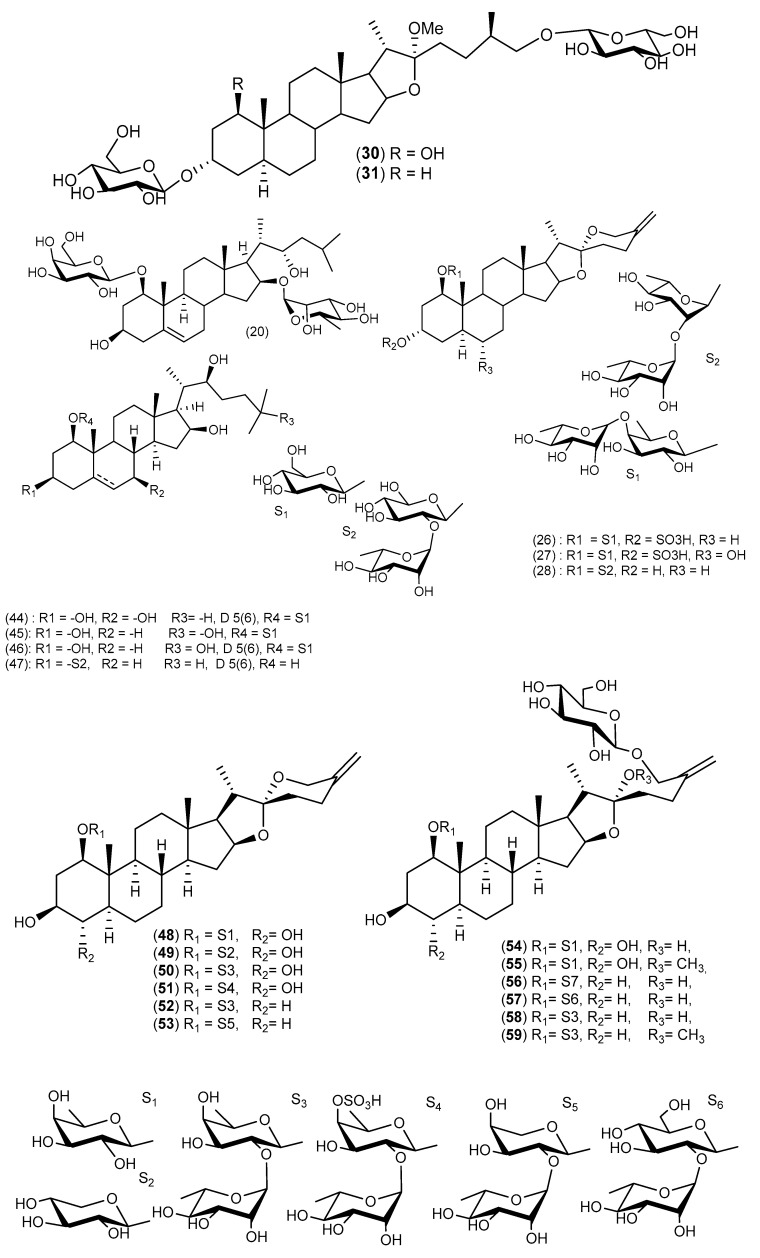

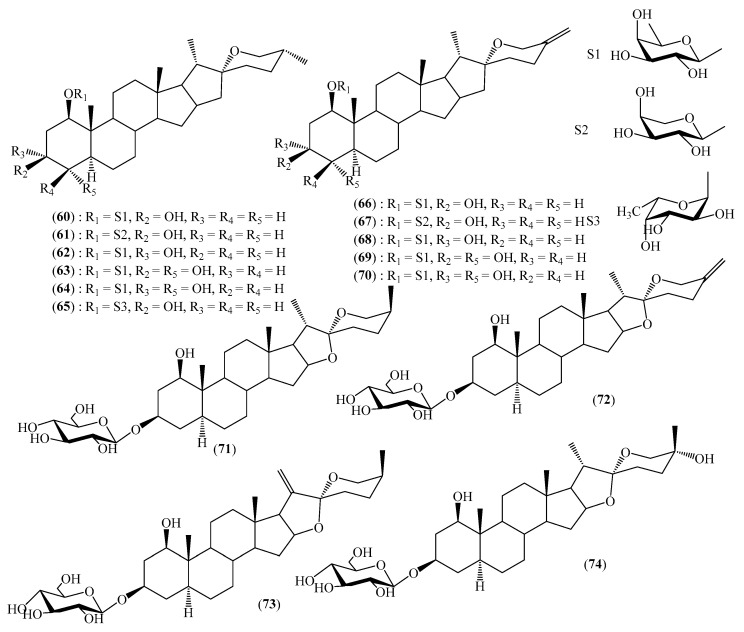

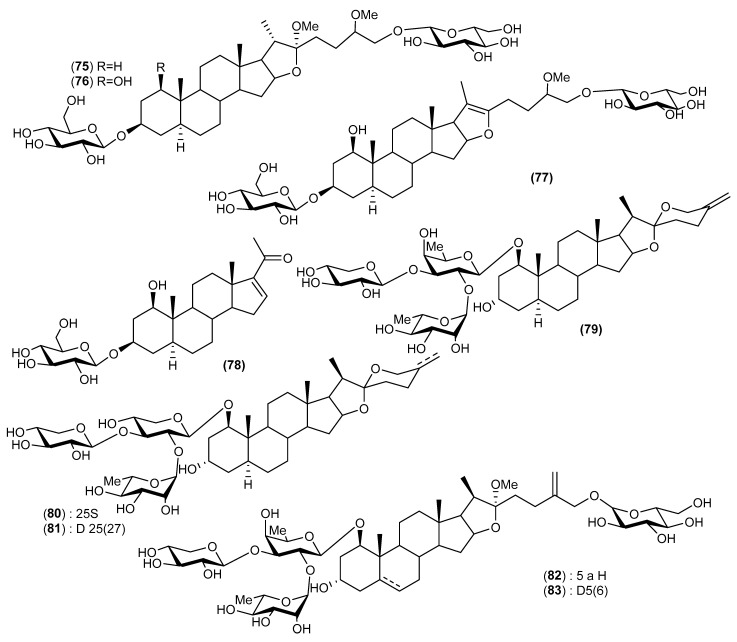
Structures of steroidal saponins isolated from some plants of the genus *Cordyline*.

**Figure 3:**
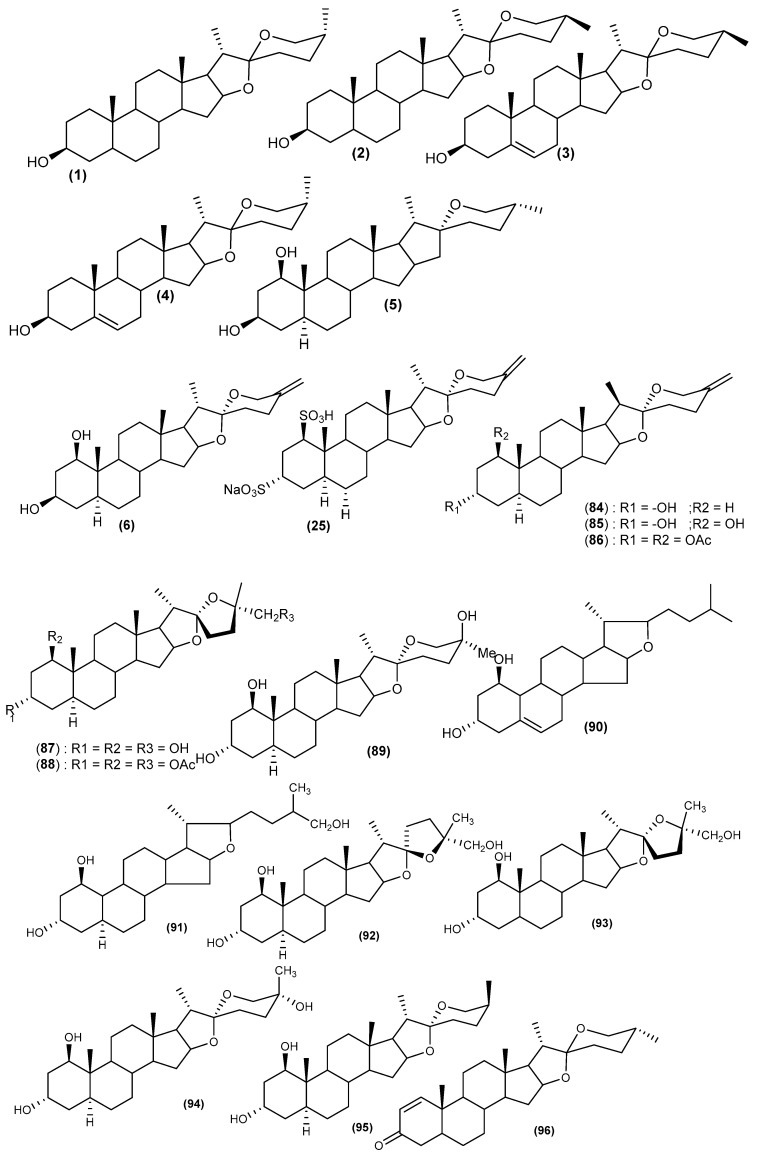
Main sapogenins isolated from plants of the genus *Cordyline*.

**Figure 4:**
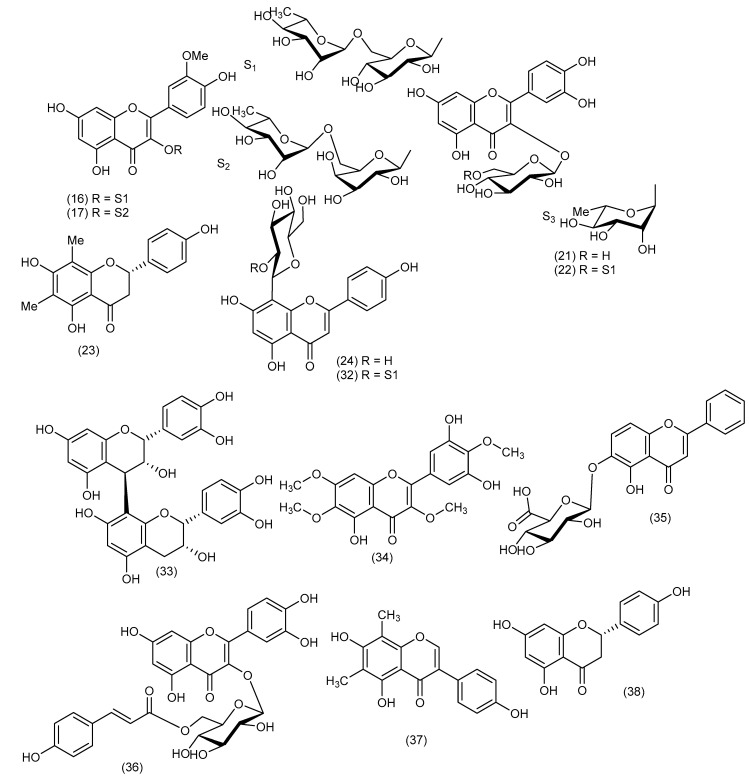

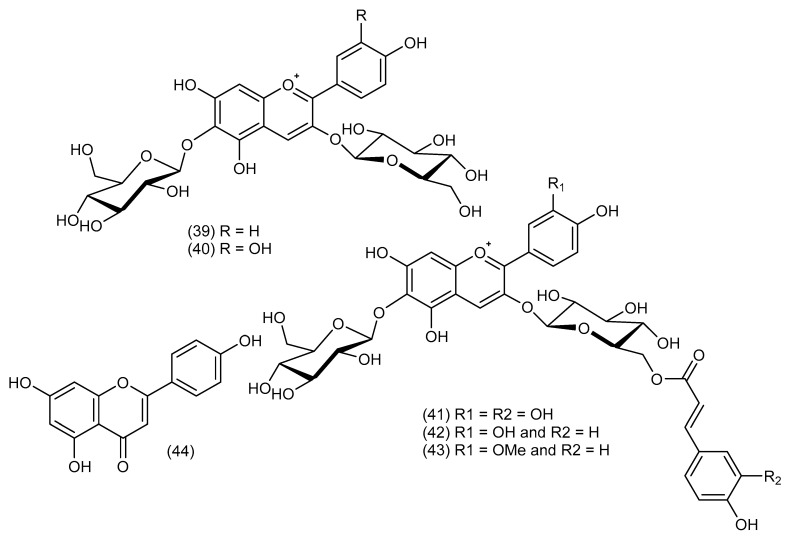
Flavonoids isolated from some plants of the genus *Cordyline*.

**Figure 5:**
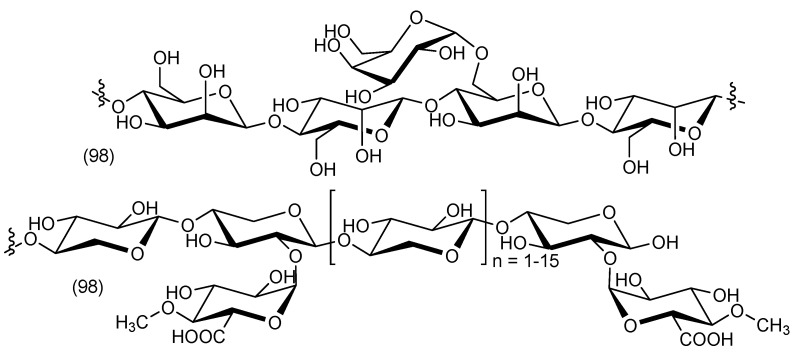
Polyosides from *Cordyline indivisa*.

A thorough review of the literature, based on the widespread uses of different parts of the plants of the *Cordyline* genus in traditional medicine, has resulted in considerable chemical analysis of their active compounds. A bibliographic investigation has revealed the isolation and identification of two principal classes of compounds with various structural skeletons. The process of isolating some of these compounds was carried out using simple column chromatography. In contrast, others used vacuum liquid chromatography on reversed-phase RP-18 silica gel medium-pressure liquid chromatography on silica gel 60. The structures of isolated compounds were established based on 1D and 2D NMR data, mass spectrometry, and chemical methods. Phytochemical studies have been mainly focused on the following eight *Cordyline* species: *C. australis*, *C. fruticosa*, *C. manners-suttoniae*, *C. stricta*, *C. rubra*, *C. dracaenoides*, *C. cannifolia*, and *C. indivisa*. The structures of the isolated secondary metabolites are grouped in Figure 2, Figure 3, Figure 4 and Figure 5.

### 5.1. Cordyline australis

The first reports of the presence of steroidal sapogenins in *Cordyline* species originated in the pioneering work by Marker et al. in 1943 and Wall et al. in 1954 [46,47], who investigated natural plant sources of steroidal sapogenins such as smilagenin (**1**) and sarsasapogenin (**2**), as substrates for the commercial synthesis of steroidal hormones. Further studies concerning the distribution of steroidal sapogenins in the genus *Cordyline* were carried out [48,49,50]. Extracts of the leaves of *C. australis* obtained from plants grown in New Zealand and the United Kingdom gave a low yield of steroidal sapogenins [6]. The major compound was characterised as tigogenin or sarsasapogenin (**2**) based on TLC and IR data, while neotigogenin or smilagenin (**1**), diosgenin (**3**), yamogenin (**4**), and brisbagenin (**5**) were identified by TLC alone [51]. From the fruits of *Cordyline australis*, the new steroidal sapogenin, australigenin [5*α*-spirost-25(27)-ene-l*β*,3*β*-diol] (**6**) was isolated, and it was also later reported from *C. manners-suttoniae* [8]. Other steroidal sapogenins have been isolated and characterised, including pompeygenin (**7**) and l*β*-hydroxycrabbogenin (**8**) [12]. Korkashvili (2006) investigated the leaves and stems of *C. autralis* in partial fulfillment of a Ph.D. degree. He obtained seven steroidal glycosides (**9**–**15**) and an inseparable mixture of two flavonoid glycosides (**16** and **17**) (Figure 4) [10].

### 5.2. Cordyline fruticosa (Synonym: Cordyline terminalis)

*Cordyline fruticosa* has been reported to contain cholestane glycosides, polyphenols, flavonoids, glucofructan, tannins, phytosterols, and steroidal alkaloid saponins. Three new steroidal saponins, spirosta-5,25(27)-diene-1*β*,3*β*-diol-1-*O*-*α*-L-rhamnopyranosyl-(1→2)-*β*-D-fucopyranoside (fruticoside H) (**18**), 5*α*-spirost-25(27)-ene-1*β*,3*β*-diol-1-*O*-*α*-L-rhamnopyranosyl-(1→2)-(4-*O*-sulfo)-*β*-D-fucopyranoside (fruticoside I) (**19**), and (22S)-cholest-5-ene-1*β*,3*β*,16*β*,22-tetrol-1-*O*-*β*-D-galactopyranosyl-16-*O*-*α*-L-rhamnopyranoside (fruticoside J) (**20**) were reported from the leaves of *Cordyline fruticosa* (Figure 2) in addition to the flavonoids quercetin 3-*O-β*-D-glucopyranoside (**21**), quercetin 3-rutinoside (**22**), Farrerol (**23**) and vitexin (**24**) (Figure 4) [7].

A new sulfated steroidal derivative fruticogenin A: 1-sulfo-australigenin-3-sodium sulphate, (**25**) (Figure 3) and three new steroidal saponins, namely, fruticoside K (3-sulfo-spirostan-25(27)-ene-1*β*,3*β*-diol-1-*O*-[*α*-L-rhamnopyranosyl-(1→4)-*β*-D-fucopyranoside], (**26**), fruticoside L (3-sulfo-spirostan-25(27)-ene-1*β*,3*β*,6*α*-triol-1-*O*-[*α*-L-rhamnopyranosyl-(1→4)-*β*-D-fucopyranoside], (**27**) and fruticoside M (spirostan-25(27)-ene-1β,3α-diol-1-*O*-[α-L-rhamnopyranosyl-(1→2)-α-L-rhamnopyranoside], (**28**) were isolated from the aerial parts of *C. fruticosa* (Figure 2) [11]. Other reports have shown that these leaves contain apigenin (**29**) [52] as well as 12 other compounds, including four known saponins, namely, fruticoside M (**13**), fruticoside I (**15**), 26-*O*-*β*-D-glucopyranosyl-22-*O*-methyl-(25*S*)-5*α*-furostane-1*β*,3*α*,22*ε*,26-tetrol 3,26-*O*-*β*-D-glucopyranoside (**30**), and 26-*O*-*β*-D-glucopyranosyl-22-*O*-methyl-(22S)-5 afurostane-3*α*,22*ε*-26-triol-3-*O*-*β*-D-glucopyranoside (**31**) and eight flavonoids, Farrerol (**23**), Vitexin 2″-*O*-rhamnoside or (5,7,4′-trihydroxyflavone 2″-*O-α*-L-rhamnopyranosyl-8-*C*-*β*-D-glucopyranoside) (**32**), procyanidin B2 (**33**), 5,3′,5′-Trihydroxy-3,6,7,4′-tetramethoxyflavone (**34**), baicalein-6-*O*-*β*-glucuronopyranoside (**35**), quercetin-3-*O*-[6-trans-*p*-coumaryl]-*β*-D-glucoside (Helichrysoside) (**36**), 4′,5,7-trihydroxy-6,8-dimethylisoflavone (**37**), and naringenin (**38**) [53].

From the purple flowers of this plant, five anthocyanidins derivatives, namely, cyanidin 3,5-di-*O*-*β*-D-glucopyranoside (**39**), peonidin 3,5-di-*O*-*β*-D-glucopyranoside (**40**), cyanidin 3-*O*-*β*-D-(6″-*O-E*-p-caffeoylglucopyranoside)-5-*O*-*β*-D-glucopyranoside (**41**), cyanidin 3-*O*-*β*-D-(6″-*O-E*-p-coumaroylglucopyranoside)-5-*O*-*β*-D-glucopyranoside (**42**), and peonidin 3-*O*-*β*-D-(6″-*O-E*-p-coumaroylglucopyranoside)-5-*O*-*β*-D-glucopyranoside (**43**) have been isolated (Figure 4) [54]. Another study conducted on *Cordyline fruticosa* led to the isolation of four new cholestane glycosides, namely (22***S***)-3*β*,7*β*,16*β*,22-tetrahydroxycholest-5-en-1*β*-yl *β*-D-glucopyranoside (**44**)**,** (22*S*)-3*β*,16*β*,22,25-tetrahydroxycholest-5-en-1*β*-yl *β*-D-glucopyranoside (**45**)**,** (22***S***)-3*β*,16*β*,22,25-tetrahydroxy-5*α*-cholestan-1*β*-yl *β*-D**-**glucopyranoside (**46**)**,** and (22*S*)-16*β*,22,25-trihydroxycholest-5-en-3*β*-yl *O*-*α*-L-rhamnopyranosyl-(1→2)-*β*-D-glucopyranoside (**47**) [55].

A recent study conducted on a C*. fruticose* cultivar called ‘Fairchild red’ from Vietnam, led to the isolation of twelve previously undescribed steroidal glycosides. Their structures were elucidated as spirostanol glycosides, 5*α*-spirost-25(27)-ene-1*β*,3*β*,4*α*-triol 1-*O*-*β*-D-fucopyranoside (**48**), 5*α*-spirost-(25)27-ene-1*β*,3*β*,4*α*-triol 1-*O*-*β*-D-xylopyranoside (**49**), 5*α*-spirost-(25)27-ene-1*β*,3*β*,4*α*-triol 1-*O*-*α*-L-rhamnopyranosyl-(1→2)-*β*-D-fucopyranoside (**50**), 5*α*-spirost-(25)27-ene-1*β*,3*β*,4*α*-triol 1-*O*-*α*-L-rhamnopyranosyl-(1→2)-(4-O-sulfo)-*β*-D-fucopyranoside (**51**), 5*α*-spirost-25(27)-ene-1*β*,3*β*-diol 1-*O*-*α*-L-rhamnopyranosyl-(1→2)-*β*-D-fucopyranoside (**52**), and 5*α*-spirost-25(27)-ene-1*β*,3*β*-diol 1-*O*-*α*-L-rhamnopyranosyl-(1→2)-*α*-L-arabinopyranoside (**53**). Furostanol glycosides were also isolated as 26-*O*-*β*-D-glucopyranosyl-5*α*-furost-(25)27-ene-1*β*,3*β*,4*α*,22*α*,26-pentol 1-*O*-*β*-D-fucopyranoside (**54**), 26-*O*-*β*-D-glucopyranosyl-22*α*-methoxy-5*α*-furost-(25)27-ene-1*β*,3*β*,4*α*,26-tetrol 1-*O*-*β*-D-fucopyranoside (**55**), 26-*O*-*β*-D-glucopyranosyl-5*α*-furost-(25)27-ene-1*β*,3*β*,22*α*,26-tetrol 1-*O*-*β*-D-glucopyranoside (**56**), 26-*O*-*β*-D-glucopyranosyl-5*α*-furost-(25)27-ene-1*β*,3*β*,22*α*,26-tetrol 1-*O*-*α*-L-rhamnopyranosyl-(1→2)-*β*-D-glucopyranoside (**57**), 26-*O*-*β*-D-glucopyranosyl-5*α*-furost-(25)27-ene-1*β*,3*β*,22*α*,26-tetrol 1-*O*-*α*-L-rhamnopyranosyl-(1→2)-*β*-D-fucopyranoside (**58**), and 26-*O*-*β*-D-glucopyranosyl-22*α*-methoxy-5*α*-furost-(25)27-ene-1*β*,3*β*,26-triol 1-*O*-*α*-L-rhamnopyranosyl-(1→2)-*β*-D-fucopyranoside (**59**) (Figure 2) [56].

### 5.3. Cordyline manners-suttoniae

The chemical investigation of *C. manners-suttoniae* fruits led to the isolation and characterisation of a total of 12 secondary metabolites, 11 of which are the steroidal saponins (1*R*,3*R*,5*S*,22*S*,25*R*)-3-hydroxyspirostanyl-1-*O*-*β*-D-fucopyranoside (**60**), (1*R*,3*R*,5*S*,22*S*, 25*R*)-3-hydroxyspirostanyl-1-*O*-*β*-L-arabinopyranoside (**61**), (1*R*,3*S*,5*S*,22*S*,25*R*)-3-hydroxyspirostanyl-1-*O*-*β*-D-fucopyranoside (**62**), (1*R*,3*S*,4*S*,5*R*,22*S*,25*R*)-3,4-dihydroxyspirostanyl-1-*O*-*β*-D-fucopyranoside (**63**), (1*R*,3*R*,4*S*,5*R*,22*S*,25*R*)-3,4-dihydroxyspirostanyl-1-*O*-*β*-D-fucopyranoside (**64**), (1*R*,3*R*,5*S*,22*S*)-3-hydroxyspirost-25(27)-enyl-1-*O*-*β*-D-fucopyranoside (**65**), (1*R*,3*R*,5*S*,22*S*)-3-hydroxyspirost-25(27)-enyl-1-*O*-*β*-L-arabinopyranoside (**66**), (1*R*,3*S*,5*S*,22*S*)-3-hydroxyspirost-25(27)-enyl-1-*O*-*β*-D-fucopyranoside (**67**), (1*R*,3*S*,4*S*,5*R*,22*S*)-3,4-dihydroxyspirost-25(27)-enyl-1-*O*-*β*-D-fucopyranoside (**68**), and (1*R*,3*R*,4*S*,5*R*,22*S*)-3,4-dihydroxyspirost-25(27)-enyl-1-*O*-*β*-D-fucopyranoside (**69**) trivially named suttonigenins A-J, respectively, were reported for the first time, one reported brisbagenin 1-*O*-*β*-D-fucopyranoside (**70**) and one reported sapogenin (**6**) (Figure 2 and Figure 4) [8].

### 5.4. Cordyline stricta

Phytochemical studies have been carried out on the leaves of *C. stricta* that resulted in the identification of four new spirostanol saponins, namely 25*S*-5*α*-spirostane-1*β*, 3*α*-diol 3-*O*-*β*-D-glucopyranoside (**71**), 5*α*-spirost-25(27) ène-1*β*,3*α*-diol 3-*O*-*β*-D-glucopyranoside (**72**), 25*S*- 5*α*-spirost-20- ene-1*β*,3*α*-diol 3-*O*-*β*-D-glucopyranoside (**73**), and 25*R*-5*α*-spirostane-1*β*, 3*α*,25-triol 3-*O*-*β*-D-glucopyranoside (**74**), three new furostanol saponins, 26-*O*-*β*-D-glucopyranosyl-22-*O*-methyl-25*S*-5*α*-furostane-3*α*-22γ-26 triols 3-*O*-*β*-D-glucopyranoside (**75**), 26-*O*-*β*-D-glucopyranosyl-22-*O*-methyl-25*S*-5*α*-furostane-1*β*-3*α*-22γ-26 tetrol 3-*O*-*β*-D-glucopyranoside (**76**), and 26-*O*-*β*-D-glucopyranosyl-5*α*-furost-20 (22)-ene1*β*-3*α*-26 triol 3-*O*-*β*-D-glucopyranoside (**77**), and a new pregnane glucoside 1*β*-3*α*-dihydryoxy-5*α*-pregn-16-en-20-one-3-*O*-*β*-D-glucopyranoside (**78**) (Figure 2) [9]. In another study, three new spirostanol saponins and two new furostanol saponins were isolated from the fresh leaves of *C. stricta*. Two of the isolated saponins contained a new branched triglycoside moiety assigned as *O*-*α*-L-rhamnopyranosyl-(1→2)-*O*-[*β*-D-xylopyranosyl-(1→3)]-*β*-D-xylopyranose with the formation of an *O*-glycosidic linkage to C-1 of the aglycone; namely 5*α*-spirost-25(27)-ene-1*β*, 3*α*- diol 1-*O*-{*O*-*α*-L-rhamnopyranosyl-(1, 2)-*O*-[*β*-*O*- xylopyranosyl-(1,3)]-*β*-D-fucopyranoside (**79**), (25*S*)-5*α*-spirostane- 1*β*, 3*α*-diol 1- *O*-{*O*-*α*-L-rhamnopyranosyl-(1, 2)-*O*-[*β*-D-xylo- pyranosyl-(1, 3)]-*β*-*O*-xylopyranoside (**80**), 5*α*-spirost-25(27)-ene-1*β*,3*α*-diol 1-*O*-{*O*-*α*-L-rhamnopyranosyl-(1→2)-*O*-[*β*-D-xylopyranosyl-(1→3)]-*β*-D-xylopyranoside} (**81**), 26-*O*-*β*-D-glucopyranosyl-22-*O*-methylfurosta- 5,25(27)-diene- 1 *β*,3*β*, 22*α*,26-tetrol 1-*O*-{*O*-*α*-L-rhamnopyranosyl-(1→2)-*O*-[*β*-D-xylopyranosyl-(1→3)]-*β*-*O*-fucopyranoside (**82**), and 26-*O*-*β*-D-glucopyranosyl-22-*O*-methylfurosta-5,25(27)-diene-1*β*,3*β*,22ξ,26-tetrol 1-*O*-{*O*-*α*-L-rhamnopyranosyl-(1→2)-*O*-[*β*-D-xylopyranosyl-(1→3)]-*β*-*O*-fucopyranoside} (**83**) (Figure 2) [4,57]. Further investigations on *Cordyline stricta* leaves have led to the isolation of four new steroidal sapogenins, crabbogenin {5*α*-spirost-25(27)-en-3*α*-ol} (**84**), 1*β*-hydroxycrabbogenin (**85**), 1-*β*-hydroxycrabbogenin diacetate (**86**), strictagenin {(20*S*, 22*S*, 25*S*)-5a-furostan-22, 25-epoxy-l*β*,3*α*-26-triol} (**87),** strictagenin triacetate (**88**) and pompeygenin ]{(25S)-5*α*-spirostane-1*β*,3*α*-25-triol} (**89**) (Figure 3) [6,51].

All the steroidal saponins presented here align with the results reported from studies on the Agavaceae family. In fact, this family is known to produce mainly steroidal saponins, as reviewed by Boyce and Tinto in 2006 [58]. In addition, one of the most widely studied genus of this family (the *Agave*) is reported to mostly contain steroidal saponins and sapogenins [59], and we noticed that the structures with Δ^25(27)^ are even more common in the genus *Cordyline* than in the genus *Agave*. The genus *Yucca* is also reported to produce such compounds [58]. This review presented several flavonoids isolated from plants of the genus *Cordyline*, while some research only highlighted the flavonoid profiles in the genus *Agave* [60].

### 5.5. Cordyline rubra

From the leaves of *C. rubra*, two new steroidal sapogenins 1*β*, 3*α*- dihydroxy-furost-5-ene (**90**) and 1*β*, 3*α*, 26- trihydroxy-5*α*-furostane (**91**) (Figure 3) [61] and three new steroidal sapogenin triols were isolated and identified as (22*S*,25*R*)-5*α*-furostan-22,25-epoxy-1*β*,3*α*,26-trio1 (rubragenin) (**92**), (22*R*,25*R*)-5*α*-furostan-22,25-epoxy-l*β*,3*α*,26-triol (wallogenin) (**93**), and (22*R*,25*R*)-5*α*-spirostane-l*β*, 3*α*,25-triol (chenogenin) (**94**). Wallogenin is epimeric at C-22 to all the previously reported ‘furanose’ F-ring sapogenins. (Figure 3) [62].

### 5.6. Cordyline cannifolia

The first chemical study on *C. cannifolia* was undertaken in 1974 by Jewers et al. Cordylagenin (**95**) (1*β*,3*α*-dihydroxy-5*α*,22*α*,25*β* spirostane), a new steroidal sapogenin diol, was isolated from the leaves [48]. Other steroidal sapogeninins from the leaves of C. cannifolia and C. stricta were also reported. The sapogenin was obtained and identified as 1*β*,3*α*-dihydroxy-5*α*,25*β*-spirostane, brisbagenin (**5**), (Figure 3) [63].

### 5.7. Cordyline dracaenoides

Chemical analysis of *C. dracaenoides* revealed the presence of steroidal saponins and anthocyanidins in the stem, rhizome and root. Small amounts of tannins were also detected in its stem and rhizome. Other studies conducted on this plant have shown the existence of steroidal saponins, including brisbagenin (**5**) and brisbenone (**96**) [63,64].

### 5.8. Cordyline indivisa

The compound 4-*O*-methylglucuronoxylan (**97**) and a very small amount of a glucomannan (**98**) (Figure 5) have been isolated from the trunk of the tropical liliaceous tree *C. indivisa* (Steud.) [65]. 

The following figures summarise all reported compounds from the *Cordyline* genus.

## 6. Biological Activities

Phytochemical investigations have revealed the presence of several constituents belonging to different classes of compounds. Due to their antibacterial, antifungal, antiparasitic, and antioxidant activities, the genus *Cordyline* has mainly been investigated for its properties already observed in the natural environment. Bioactivities reported for *Cordyline* plant extracts and their pure compounds are summarised by activity in this section.

### 6.1. Antimicrobial Activity

The antimicrobial activity of fruticoside H (**18**), fruticoside I (**19**), and fruticoside J (**20**) was assessed against *Staphylococcus aureus, Escherichia coli, Pseudomonas aeruginosa, Enterococcus faecalis,* and *Candida albicans*. The three isolated saponins, even at the highest concentration used in the test (256 mg/L), were not able to inhibit growth except for compound **19** (Fructicoside I) against *E. faecalis*. In this case, the minimum inhibitory concentration (MIC) value was 128 mg/L, which, although still high, is indicative of a specific antibacterial activity. The minor structural difference between **18** and **19** suggested that the presence of the SO_3_H group at C-4’ and the absence of the double bond at C-5(∆^5,6^) can significantly decrease the MIC value (more than 256 μg/mL for **18** and 128 μg/mL for **19**) of the molecules against the enterococcal species [7]. The *n*-hexane, ethyl acetate, and methanol extracts of *C. fruticosa* were evaluated for their antibacterial capacity against Gram-negative *Escherichia coli* and *Salmonella typhi* as *Staphylococcus aureus* and Gram-positive *Bacillus subtilis* by the microdilution method. The ethyl acetate fraction was found to be the most active against *Salmonella typhi* (inhibition diameter ID 15.2 mm) and *S. aureus* (ID 14.2 mm) in comparison to penicillin (ID 83.5 and 73.2 mm), tetracycline (ID 76.8 and 72.1 mm) and streptomycin (ID 77.2 and 71.4 mm) used as standards [14]. The crude methanol extract of *C. fruticosa* showed a faint zone of inhibition against *E. fecalis* [13]. Firoj et al. (2003) reported antibacterial activity in methanol, chloroform, acetone, and hexane extracts of *Cordyline fruticosa.*

Moderate antibacterial activity was observed for methanol extracts against *Shigella boydii*, *Staphylococcus epidermis*, and *Streptococcus pyogenes*, with inhibition zone values of 14, 12, and 13 mm, respectively, in comparison to Kanamycin (38, 32, and 40, respectively). Hexane extracts have exhibited mild antibacterial activity against *Escherichia coli*, *Salmonella typhi*, *Shigella boydii*, and *Shigella dysenteriae* (with inhibition zone values of 8, 9, 8, and 9 mm, respectively) relative to 39, 30, 38, and 39 mm showed by Kanamycin. In contrast, acetone and chloroform extracts showed no significant activities [66].

Prihambodo et al. (2019) investigated the antibacterial potential of *C. fruticosa*. They found that the MeOH extract exhibited interesting activity against *Escherichia coli* and *Salmonella tyhpimurium*, with an inhibitory area of 6.72 ± 0.95 mm and 6.52 ± 1.21 mm, respectively, indicating that this extract possesses medium antibacterial activity (5–10 mm) [67]. *n*-Hexane, ethyl acetate, and methanol extracts were also investigated for their potential antibacterial activity by Elfita et al. (2019). The methanol extract displayed the highest antibacterial activity using the Kirby–Bauer method against Gram-negative (*Salmonella typhi*, ID 15.1 mm; *Escherichia coli*, ID 16.7 mm) and Gram-positive (*Staphylococcus aureus*, ID 17.0 mm; *Bacillus subtilis*, ID 12.5 mm) compared to 19.4, 19.5, 21.1, and 20.6 mm, respectively, for tetracycline [68].

The anticandidal activity of *C. fruticosa* leaf extracts was examined using agar well diffusion against *Candida albicans*. *N*-hexane, ethyl acetate, and ethanol extracts showed weak inhibition zones of 13, 8 and 12 mm, respectively, at a concentration of 400 μg/well, compared to 100 mm of the Chloramphenicol [69].

Antibacterial activities were assayed for fruticoside H (**18**), fruticoside I (**19**), and fruticoside J (**20**). Of these, only fruticoside H (**18**) displayed moderate antibacterial activity against *Enterococcus faecalis* (MIC = 128 μg/mL) [7]. The isolated compounds from the fruit of *Cordyline manners-suttoniae* (**6**, **60**–**69**) were assayed for their antibacterial activity by using a microdilution method against *Enterococcus faecium* (ATCC 35667 and C15), *Staphylococcus aureus* (ATCC 25923 and ATCC 29247), *Klebsiella pneumoniae* (ATCC 13883 and ATCC 12657), *Acinetobacter baumannii* (ATCC 19606 and ATCC 17978), *Pseudomonas aeruginosa* (ATCC 10145 and ATCC 49189), and *Enterobacter aerogenes* (ATCC 13048 and ATCC 13047). Suttonigenin A (**60**), suttonigenin C (**62**), suttonigenin D (**63**), suttonigenin E (**64**), suttonigenin G (**66**), suttonigenin H (**67**), suttonigenin I (**68**), and suttonigenin J (**69**) were found to inhibit the growth of the Gram-positive bacteria (*S. aureus* and *E. faecium*) with MIC_75_ values ranging from 5.4 to 84.3 μM relative to 9.7 μM of Chloramphenicol (positive control). A dichloromethane extract also displayed antibacterial activity against these strains [8].

### 6.2. Antioxidant Activity

Antioxidant activity measured using the 2,2-diphenyl-1-picryl-hydrazyl (DPPH) method of crude extract of *C. fruticosa* purple flowers and isolated compounds showed the potential to scavenge free radicals. In fact, the methanol crude extract displayed an IC_50_ of 13.1 μg/mL, while isolated compounds (**39**–**43**) showed IC_50_ in the range of 13.8 to 16.4 μg/mL compared to that of quercetin (4.5 μg/mL) used as a positive control [54]. Antioxidant activity of encapsulated (with sodium caseinate beads) and unencapsulated phenolic content of *C. fruticosa* methanolic extract were assessed for their free radical scavenging capacity per gram of ascorbic acid equivalent (AAE). The encapsulated extracts showed good antioxidant activity after one year of conservation (EC_50_ 27.38 mg/g AAE) in comparison to the unencapsulated extracts (EC_50_ 13.4 mg/g AAE) [70]. A similar method was investigated on the methanol extract of *C. fruticosa* leaves; this extract was combined with copper oxide to form nanoparticles. Both crude extract and nanoparticles exhibited antioxidant activity following the DPPH method, with ascorbic acid used as a positive control [71]. As has been reported with several medicinal plants [72,73], this antioxidant activity is linked to the flavonoid content of *Cordyline fruticosa*.

### 6.3. Antiproliferative Activity

The antiproliferative effect of steroidal saponins is well reported and is known to affect cancer cell lines following several mechanisms such as apoptosis, differentiation, senescence, autophagy, oncosis, and other pathways [74,75,76]. The effect of fruticoside H-J (**18**–**20**) on the viability of the MDA-MB 231 human breast adenocarcinoma cell line, HCT 116 human colon carcinoma cell line and A375 human malignant melanoma cell line were evaluated in vitro using the MTT assay. Fruticoside H and I (**18** and **19**) showed moderate antiproliferative activity, with IC_50_ values ranging from 37.83 to 69.68 µM for **18** on A375 and MDA-MB 231 cells, respectively, and from 46.59 to 59.97 µM for **19** on A375 and HCT116 cells, respectively. Fruticoside J (**20**) was inactive on the three tumour cell lines tested [7]. Using the same method against the same cell lines, investigators found that the steroidal saponins Fruticoside K and M (**26** and **28**) displayed weak cytotoxicity against melanoma (A375), breast adenocarcinoma (MDA-MB-231), and colon carcinoma (HCT116) human tumour cell lines [11].

Cytotoxicity of compounds **6**, **60**, and **69** was also evaluated against the cancer cell lines of human keratinocytes and human dermal fibroblasts. They all exhibited cytotoxicity with IC_50_ values ranging from 18.2 to 74.9 μM for human keratinocyte cells and 4.2 to 16.5 μM for human dermal fibroblast cells [8].

Compounds **49**–**59** were evaluated for their cytotoxicity using the MTS method [3-(4,5-dimethylthiazol-2-yl)-5-(3-carboxymethoxyphenyl)-2-(4-sulfophenyl)-2H-tetrazolium] against the 4T1 cell line from a mouse mammary gland tissue, an animal model for stage IV human breast cancer. Compounds **49**, **54**, and **55** displayed good cytotoxic activity with the respective IC_50_ values of 1.39, 1.52, and 3.24 μM, compared to 5-FU (IC_50_ 6.70 Μm) [58]. The results obtained for **49**, **54**, **55**, and **69** seem to contradict those of Zhu et al. (2015), who reported that anticancer activity increased with the number of sugars. Several compounds tested with a high number of sugars exhibited low activity, and it is possible that the aglycone moieties could have contributed to this activity (likely the double bonds Δ^5−6^ and Δ^25(27)^). Additionally, the hydroxyl at C-3 could have had an effect [77]. This is in alignment with compound **20**, which had no activity, and the presence of the double bond Δ^5−6^ and the absence of the double bond Δ^25(27)^) could explain this inactivity.

### 6.4. Hypolipidemic Potential, Antiparasitic Activity, Nephrotoxicity and Hepatotoxicity Preventions

The methanol extract of the rhizomes of *C. fruticosa* has been investigated for its hypolipidemic potential in Wistar rats. Intake of 30 mg/Kg and 70 mg/Kg was found to lower lipid plasma levels in mice [78]. This extract showed the potential to lower blood fatty acid, glucose, and triglyceride levels in obese Wistar rats [79]. The *n*-butanol-soluble fraction of the methanol extract of senescent leaves of *C. australis* was assessed for its antiparasitic activity; it displayed a weak inhibition against *Plasmodium falciparum*, *Tripanosoma brucei*, and *Tripanosoma cruzi*, with an IC_50_ of 12, 23 and 32 µM, respectively, relative to 0.018 µM of Chloroquine [10]. The aqueous extract of *C. fruticosa* also displayed antitrypanosomial activity by slowing the mitotic cycle in *Trypanosoma evansi*, with an IC_50_ of 48.1 µg/mL [80].

The effect of *C. fruticosa* leaf extract against doxorubicin-cisplatin-induced nephrotoxicity and hepatotoxicity in rats was assessed. Administration of *C. fruticosa* extract induced a significant decrease in the aspartate aminotransferase, alanine amino transferase, alkaline phosphatase, urea and creatinine in doxorubicin-treated rats. It protected the renal tissues from necrosis induced by doxorubicin. The administration of the extract 10 days before cisplatin ingestion in the preventive process contributed to a pronounced protection against hepatic injury induced by cisplatin [55].

The effect of *C. fruticosa* leaves’ methanol extract was studied on castor oil-induced diarrhoea and magnesium sulphate-induced diarrhoea. The extract significantly reduced the frequency of magnesium sulphate-induced diarrhoea at the dose of 800 mg/Kg of body weight [81].

Based on the number of studies that have reported the biological activities of steroidal saponins, we recommend that future biological assays address their effect on inflammatory cells, nervous cells, and fungi. The study of the action mechanism of the double bond Δ^25(27)^ could be carried out to shed light on their effect on cancer cells.

## 7. Toxicology

In a brine shrimp lethality test after 24 h, surviving brine shrimp nauplii were counted, and LC50 was assessed. The methanol extract of *C. fruticosa* exhibited considerable toxicity, with an LC50 value of 355.7 μg/mL, compared to a standard of vincristine sulfate (LC50 of 3.8 μg/mL). This result indicates that the methanol extract has a cytotoxicity effect and it could have antitumour molecules [81].

## 8. Conclusions

Due to their versatile traditional uses, *Cordyline* species have been extensively studied for their phytoconstituents as well as for their biological activities. Flavonoid, spirostane, furostane, and cholestane glycosides have been isolated from seven *Cordyline* species, while *C. indivisa* remains largely unstudied. In summary, the plant extracts and some of their pure compounds possess a broad spectrum of biological activities such as antibacterial, antifungal, antiproliferative, antiparasitic, and hypolipidemic effects. Saponins, sapogenins, and flavonoids are the most frequently isolated and identified classes of compounds in the species *C. australis*, *C. fruticosa*, *C. manners-suttoniae*, *C. stricta*, *C. rubra*, *C. cannifolia*, and *C. dracaenoides*, and currently constitute the only investigated and identified phytochemicals. *Cordyline* plants have been and continue to be used in traditional medicine against certain infections caused by bacteria, fungi, and parasites. This review of all available literature reveals that steroidal saponins, steroidal sapogenins, and flavonoids are the biomarkers of plants belonging to the *Cordyline* genus and present in one view the wide opportunities for the use of *Cordyline* plants in drug discovery with regard to bioactive molecules not yet characterised from this genus. The bioassay-guided isolation and identification of the bioactive components of plants of the genus *Cordyline* remain un-investigated, and more detailed in silico studies are required to propose detailed structure–activity relationships in order to determine the mechanisms of action of saponin and flavonoid constituents.

Although some compounds isolated from plants of the genus *Cordyline* have exhibited interesting biological activities, much more work is needed to exploit them in the pharmaceutical industry, as they were generally obtained in small amounts. Synthesis of some of these secondary metabolites from readily available reactants should be considered by pharmacochemists in order to obtain amounts sufficient for use in drug development.

## Data Availability

No new data were created or analysed in this study. Data sharing is not applicable in this article.

## References

[1] Porter R.J., Medrum B.S., Katzung B.G., Masters S.B., Trevor A.J. (1996). Antiseizure drug. Basic & Clinical Pharmacology.

[2] Muazu J., Kaita A.H. (2008). A review of traditional plants used in the treatment of epilepsy amongst the Hausa/Fulani tribes of northern Nigeria. Afr. J. Tradit. Complement. Altern. Med..

[3] Newman D.J., Cragg G.M. (2012). Natural products as sources of new drugs over the 30 years from 1981 to 2010. J. Nat. Prod..

[4] Cragg G.M., Pezzuto J.M. (2016). Natural products as a vital source for the discovery of cancer chemotherapeutic and chemopreventive agents. Med. Princ. Pract..

[5] Sofi M.S., Sateesh M.K., Bashir M., Ganie M.A., Nabi S. (2018). Chemopreventive and anti-breast cancer activity of compounds isolated from leaves of *Abrus precatorius* L. Biotech.

[6] Blunden G., Jaffer A.J., Jewers J.K., Griffin W.J. (1981). Steroidal sapogenins from leaves of *Cordyline* species. J. Nat. Prod..

[7] Fouedjou R.T., Teponno R.B., Quassinti L., Bramucci M., Petrelli D., Vitali L.A., Fiorini D., Tapondjou L.A. (2014). Steroidal saponins from the leaves of *Cordyline frutcosa* (L) A. Chev and their cytotoxic and antimicrobial activity. Phytochem. Lett..

[8] Tran T.D., Olsson M.A., Choudhury M.A., McMillan D., Cullen J., Parsons P.J., Bernhardt V.P., Reddell P.W., Ogbourne M.S. (2019). Antibacterial 5*α*-Spirostane saponins from the fruit of *Cordyline manners-suttoniae*. J. Nat. Prod..

[9] Mimaki Y., Kuroda M., Akaashi Y., Sashida Y. (1997). Steroidal glucosides from leaves of *Cordyline stricta*. Phytochemistry.

[10] Korkashvili T. (2006). Steroidal glycosides of *Cordyline australis*. Master’s Thesis.

[11] Ponou K.B., Teponno B.R., Tapondjou A.L., Lacaille-Dubois M., Quassinti L., Bramussi M., Barboni L. (2019). Steroidal saponins from the aerial parts of *Cordyline fruticosa* L. Var strawberries. Fitoterapia.

[12] Blunden G., Sitton D., Beach S., Turne C.H. (1984). Australigenin, A new steroidal sapogenin from *Cordyline australis* fruits. J. Nat. Prod..

[13] Chunduri J.R., Shah H.R. (2016). FTIR phytochemical fingerprinting and antioxidant analysis of selected indoor non-flowering indoor plants and their industrial importance. Int. J. Curr. Pharm. Res..

[14] Elfita M., Muharni, Mardiyanto, Fitrya (2021). Chemical compounds from the antibacterial active fraction of *Cordyline fruticosa* (L). Conf. Series: Earth Environ. Sci..

[15] Encyclopedia Britannica Plant-Nomenclature-Taxonomy. https://www.britannica.com/biography/Michel-Adanson.

[16] Mckenzie E.H.C., Buchanan P.K., Johnston P.R. (2005). Checklist of fungi on cabbage trees (*Cordyline* spp.) and New Zealand flaxes (*Phormium* spp.) in New Zealand. N. Z. J. Bot..

[17] Harris W., Ross E., Beever E.R., Heenan B.P. (1998). Phenotypic variation of leaves of and stems of wild stands of *Cordyline autralis* New *Zealand J*. New Zealand J. Bot..

[18] Ehrlich C. (1999). The Ethnobotany of *Cordyline fruticosa* (L.) A. Chev.: The “Hawaiian Ti plant”. Ph.D. Dissertation.

[19] Khan S., Naz S., Saeed B. (2004). In vitro production of *Cordyline terminalis* for commercialization. Pak. J. Bot..

[20] Kattoor C.J. (2010). Micropropagation Studies in *Dracaena* and *Cordyline*. Master’s Thesis.

[21] Wilson A. (1994). Flora of Australia: Oceanic Islands, 49.

[22] Conran J.G., Kubitzki K. (1998). Lomandraceae. The Families and Genera of Vascular Plants.

[23] World Checklist of Selected Plant Families (WCSP). https://wcsp.science.kew.org/.

[24] Chifundera K. (1998). Livestock diseases and the traditional medicine in the Bushi area. Kivu province, Democratic Republic of Congo. Afr. Study Monogr..

[25] Lense O. (2012). The wild plants used as traditional medicines by indigenous people of Manokwari, West Papua. Biodiversitas.

[26] Calixto J.B., Lima T.C.M., Morato G.S., Nicolau M., Takahashi R.N., Valle R.M.R., Schmidt C.C., Yunes R.A. (1990). Chemical and pharmacological analysis of the crude aqueous/alcoholic extract from *Cordyline dracaenoides*. Phytother. Res..

[27] Melzer M., Sugano J., Uchida J., Kawate M., Borth W., Hu J. Partial characterization of a novel emara-like virus from *Cordyline fruticosa* (L.) with ti ringspot disease. Proceedings of the 2014 APS-CPS Joint Meeting.

[28] Hinkle A.E. (2007). Population structure of pacific *Cordyline fruticosa* (Laxmanniaceae) with implications for human settlement of Polynesia. Am. J. Bot..

[29] Whistler W.A. (1985). Traditional and herbal medicine in the Cook Islands. J. Ethnopharm..

[30] Buttner R., Hanelt P. (2001). Mansfeld’s Encyclopedia of Agricultural and Horticultural Crops.

[31] Dalimartha S. (2007). Atlas Tumbuhan Obat Indonesia.

[32] Nugent J. (2006). Permaculture Plants: Agaves and Cacti.

[33] Nombo P., Leach J. (2010). Reite Plants: An Ethnobotanical Study in Tok Pisin and English.

[34] Kulip J. (2003). An ethnobotanical survey of medicinal and other useful plants of Muruts in Sabah, Malaysia. Telopea.

[35] Kent H.W. (1995). Treasury of Hawaiian Words in One Hundred and One Categories. The Masonic Public Library of Hawaii.

[36] Ray T., Saha P., Roy S.C. (2006). Commercial production of *Cordyline terminalis* (L) Kunth. From shoot apex meristem and assessment for genetic stability of somaclones by isozyme markers. Sci. Hortic..

[37] Brasch D.J., Fankhauser B.L., McDonald A.G. (1988). A study of the glucofructofuranon from the New Zealand cabbage tree *Cordyline australis*. Carbohydr. Res..

[38] Simpson P. (2000). Dancing Leaves. The Story of New Zealand’s Cabbage Tree, TıKouka.

[39] Aufenanger H. (1961). The *Cordyline* plant in the central highlands of new Guinea. Anthropos.

[40] Merlin M. (1989). The traditional geographical range and ethnobotanical diversity of *Cordyline fruticosa* (L.) Chevalier. Ethnobotany.

[41] Whistler W.A. (1992). Polynesian Herbal Medicine. Lawa’i, Kaua’i, Hawai’i.

[42] Elbert L.L.J., Roger G.S. (1989). Common Forest Trees of Hawaii (Native and Introduced).

[43] Lim T.K. (2015). Edible Medicinal and Non-medicinal Plants. Volume 12: Modified Stems, Roots, Bulbs.

[44] Duncalf I. (2014). Conventional propagation of Cordyline australis. Act. Hortic..

[45] Simpson P. (1991). Sudden decline in cabbage trees (*Cordyline australis*). Sci. Res. Intern. Rep..

[46] Marker R.E., Wagner R.B., Ulshafer P.R., Wittbecker E.L., Goldsmith D.P.J., Ruof C.H. (1943). Sterols. CLVII. Sapogenins. 69. Isolation and structures of thirteen new steroidal sapogenins. New sources for known sapogenins. J. Am. Chem. Soc..

[47] Wall M.E., Eddy C.R., Willaman J.J., Correll D.S., Schubert B.G., Gentry H.S. (1954). Steroidal sapogenins. XII. Survey of plants for steroidal sapogenins and other constituents. J. Am. Pharm. Assoc..

[48] Jewers A.H.K., Manchanda J.D., Nagle M.J. (1974). Cordylagenin, a new steroidal saponin diol from *Cordyline cannifolia* and *Cordyline stricta*. Tetrahedron Lett..

[49] Blunden G., Jaffer J.A., Jewers K., Robinson J.M. (1980). Configurational assignments to spirostan-3-ols by mass spectrometry. Steroids.

[50] Griffin W.J., Maunwongyathi P. (1969). A comparison of four species of *Cordyline*. Planta Med..

[51] Blunden G., Jaffer J.A., Jewers K., Griffin W.J. (1981). Crabbogenin 1*β*-hydroxycrabbogenin, strictagenin and pompeygenin, four new steroidal sapogenins from *Cordyline stricta* leaves. Tetrahedron.

[52] Djohan H., Widiyantoro A., Shofiyani A. (2019). Isolation of flavonoid from Andong leaves (*Cordyline fruticosa* (L.) A. Chev.) and its activity as complexor of Fe^2+^. Indones. J. Pure Appl. Chem..

[53] Raslan M., Taher R.F., Al-Karmalawy A.A., El-Ebeedy D., Metwaly A.G., Elkateeb N.M., Ghanem A., Elghaish R.A., El Maksoud A.I.A. (2021). *Cordyline fruticosa* (L.) A. Chev. leaves: Isolation, HPLC/MS profiling and evaluation of nephroprotective and hepatoprotective activities supported by molecular docking. New J. Chem..

[54] Adaku C., Skaar I., Byamukama R., Jordheim M., Andersen Ø.M. (2020). Anthocyanin profile and antioxidant property of anti-asthma flowers of *Cordyline terminalis* (L.) Kunth (Agavaceae). Nat. Prod. Commun..

[55] Yokosuka A., Suzuki T., Mimaki Y. (2012). New cholestane glycosides from the l eaves of *Cordyline terminalis*. Chem. Pharm. Bull..

[56] Nguyen D.H., Mitaine-Offer A.C., Miyamoto T., Tanaka C., Bellaye P.S., Collin B., Chambin O., Lacaille-Dubois M.A. (2021). Steroidal glycosides from the Vietnamese cultivar *Cordyline fruticosa* “Fairchild red”. Phytochemistry.

[57] Yang M., Blunden G., Patel A., Crabb T.A., Griffint W.J. (1990). Two furostane sapogenins from *cordyline rubra*. Phytochemistry.

[58] Simmons-Boycea J.L., and Tinto W.F. (2006). Steroidal saponins and sapogenins from the Agavaceae family. Nat. Prod. Commun..

[59] Sidana J., Singh B., Sharma O.P. (2016). Saponins of *Agave*: Chemistry and bioactivity. Phytochemistry.

[60] Almaraz-Abarca N., Delgado-Alvarado E.A., Ávila-Reyes J.A., Uribe-Soto J.N., González-Valdez L.S. (2013). The phenols of the genus *Agave* (Agavaceae). J. Biomater. Nanobiotechnol..

[61] Mimaki Y., Kuroda M., Akaashi Y., Sashida Y. (1998). Steroidal saponins from the leaves of *Cordyline stricta*. Phytochemistry.

[62] Jewers K., Burbage M.B., Blunden G., Griffin W.J. (1974). Brisbagenin and brisbenone, two new spirostanes from *Cordyline* species. Steroids.

[63] Griffin W., Blunden G., Jewers K., Burbage B.M., Nagler M.J. (1976). Steroidal sapogenins from *Cordyline cannifolia* leaves. Phytochemistry.

[64] Yang M., Blunden G., Patel A., Crabb T.A., Griffint W.J. (1989). Rubragenin, chenogenin and wallogenin, steroidal sapogenins from *cordyline rubra*. Phytochemistry.

[65] Sieber R. (1972). Die hemicellulosen von *Cordyline indivisa*. Phytochemistry.

[66] Firoj A., Prabir K.D., Islam M.A., Rahman K.M., Rahman M.M., Selim M.S.T. (2003). Antibacterial activity of *Cordylline terminalis Kunth* leaves. J. Med. Sci..

[67] Prihambodo T.R., Nahrowi, Jayanegara A. (2019). Antibacterial activity and phytochemical content of silage juice from tropical herbal leaves. IOP Conf. Ser. Mater. Sci. Eng..

[68] Elfita E., Mardiyanto, Fitrya, Larasati J.E., Julinar, Widjajanti H., Muharni (2019). Antibacterial activity of *Cordyline fruticosa* leaf extracts and its endophytic fungi extracts. Biodiversitas.

[69] Kusuma I.W., Sari N.M., Murdiyanto, Kuspradini H. (2016). Anticandidal activity of several plants used by Bentian tribe in East Kalimantan, Indonesia. AIP Conf. Proc..

[70] Reddy C., Noor A., Sarada N.C., Vijayalakshmi M.A. (2011). Antioxidant properties of *Cordyline terminalis* (L.) Kunth and *Myristica fragrans* Houtt. Encapsulated separately into casein beads. Curr. Sci..

[71] Sharmila D., Kumar R.S. (2019). Antioxidant potential of copper oxide nanoparticles using leaves of *Cordyline fruticosa*. Drug Invent. Today.

[72] Banjarnahor S.D.S., Artanti N. (2014). Antioxidant properties of flavonoids. Med. J. Indones..

[73] Tsakem B., Tchuenguem R.T., Siwe-Noundou X., Ponou K.B., Dzoyem J.P., Teponno R.B., Krause R.W.M., Tapondjou A.L. (2022). New bioactive flavonoid glycosides with antioxidant activity from the stem bark of *Olax subscorpioidea* Oliv. Nat. Prod. Res..

[74] Yang C.-R., Zhang Y., Jacob M.R., Khan S.I., Zhang Y.-J., Li X.-C. (2006). Antifungal activity of C-27 steroidal saponins. Antimicrob. Agents Chemother..

[75] Escobar-Sánchez M.L., Sánchez-Sánchez L., Sandoval-Ramírez J. (2015). Steroidal Saponins and Cell Death in Cancer, Chapter 15. Cell Death—Autophagy, Apoptosis and Necrosis.

[76] Sobolewska D., Galanty A., Grabowska K., Makowska-Was J., Wro’bel-Biedrawa D., Podolak I. (2020). Saponins as cytotoxic agents: An update (2010–2018). Part I—Steroidal saponins. Phytochem. Rev.

[77] Zhu L., Tan J., Wang B., Guan L., Liu Y., Zheng C. (2011). In-vitro antitumor activity and antifungal activity of pennogenin steroidal saponins from *Paris Polyphylla* var. yunnanensis. Iran. J. Pharm. Res..

[78] Bogoriani N.W., Ariati N.K. (2018). The activity of Bali Andong rhizome extract of *Cordyline Terminalis* Kunth as Hypolipidemia agent in Wistar rats with high-cholesterol diet. Int. J. Pharm. Phytopharmacol. Res..

[79] Bogoriani N.W., Suaniti N.M., Putra A.A.B., Lestari K.D.P. (2019). The activity of *Cordyline terminalis*’s leaf extract as antidiabetic in obese wistar rats. Int. J. Pharm. Res. Allied Sci..

[80] Dyary H.O., Arifah A.K., Sharma R.S.K., Rasedee A. (2014). Antitrypanosomal and cytotoxic activities of selected medicinal plants and effect of *Cordyline terminalis* on trypanosomal nuclear and kinetoplast replication. Pak. Vet. J..

[81] Naher S., Aziz A.M., Akter I., Rahman S.M., Sajon S.R., Mazumder K. (2019). Anti-diarrheal activity and brine shrimp lethality bioassay of methanolic extract of *Cordyline fruticosa* (L.) A. Chev. Leaves. Clin. Phytosci..

